# Switchable Interlayer Magnetic Coupling of Bilayer CrI_3_

**DOI:** 10.3390/nano11102509

**Published:** 2021-09-27

**Authors:** Yue Jiang, Yandong Guo, Xiaohong Yan, Hongli Zeng, Liyan Lin, Xinyi Mou

**Affiliations:** 1 College of Electronic and Optical Engineering, Nanjing University of Posts and Telecommunications, Nanjing 210046, China; jiangyue@jit.edu.cn (Y.J.); 1020020804@njupt.edu.cn (X.M.); 2 College of Science, Jinling Institute of Technology, Nanjing 211169, China; 3 Key Laboratory of Radio Frequency and Micro-Nano Electronics of Jiangsu Province, Nanjing 210023, China; hlzeng@njupt.edu.cn; 4 New Energy Technology Engineering Laboratory of Jiangsu Province, Nanjing 210046, China; 5 College of Natural Science, Nanjing University of Posts and Telecommunications, Nanjing 210046, China; 1019081819@njupt.edu.cn

**Keywords:** interlayer magnetism, stacking order, magnetism controlling, van der Waals magnet

## Abstract

Due to the weak van der Waals (vdW) interlayer interaction, interfacial geometry of two-dimensional (2D) magnetic vdW materials can be freely assembled, and the stacking order between layers can be readily controlled, such as laterally shifting or rotating, which may trigger the variation of magnetic order. We investigate the H-type bilayer CrI${}_{3}$ where the two layers are aligned in anti-parallel directions. Based on first-principles calculations, we propose two states with different interlayer magnetic couplings, i.e., ferromagnetic and antiferromagnetic, and analyze the superexchange mechanism inside. It is found that the two magnetic coupling states are stacking-dependent, and could be switched by applying out-of-plane axial strain and electron doping. Our findings show great application potential in the design of heterostructural and spintronic devices based on 2D magnetic vdW materials.

## 1. Introduction

Since the experimental fabrication of atomically thin Cr${}_{2}$Ge${}_{2}$Te${}_{6}$ [1] and CrI${}_{3}$ [2] in 2017, the two-dimensional (2D) intrinsic magnetic van der Waals (vdW) materials have attracted worldwide attention [3,4,5,6,7,8]. The monolayer of these materials can be prepared by crystal exfoliation [1,2,3,6] or molecular beam epitaxy [4,5], and they can keep the long-range spin order down to the monolayer limit, which makes them become the research focus of novel magnetic semiconductors and magnetic storage materials, and the research of spintronic devices based on 2D magnetic vdW materials has also been rapidly developed [6,9,10,11]. In addition, due to the weak interlayer vdW interaction, the interface of the 2D vdW materials can be freely assembled, and the stacking order of them can be conveniently controlled, such as laterally shifting or rotating between layers, which could bring about the changes of electronic structures or magnetism and has important applications in band engineering and the design of vdW magnetic tunneling junctions [6,10,12,13,14], etc.

Among them, the family of chromium trihalides has gained special interest. The CrBr${}_{3}$ and CrI${}_{3}$ monolayers and few-layers have been experimentally prepared. Both experiments and theoretical calculations reveal that the monolayer CrI${}_{3}$ shows intralayer ferromagnetism with an out-of-plane easy axis [2,15,16]. In the bulk CrI${}_{3}$, layers stack one by one with the same orientation. For clarity, this kind of stacking structure is called R-Type. Two phases of R-type CrI${}_{3}$ in different temperatures are reported experimentally. When heated to 210–220 K, the rhombohedral phase in low temperature turns into the monoclinic phase in high temperature [15]. Here, we denote the rhombohedral phase of bilayer CrI${}_{3}$ at a low temperature as A-phase, and the monoclinic phase at a high temperature as B-phase. Recently, it has been reported that, for the R-type bilayer CrI${}_{3}$, the A-phase shows interlayer ferromagnetic (FM) coupling while the B-phase exhibits interlayer antiferromagnetic (AFM) coupling, and the magnetism can be switched through a lateral shift between the two layers [16,17]. On the other hand, CrBr${}_{3}$ is another vdW layered semiconductor, which has a similar structure to CrI${}_{3}$. Experiments [5] demonstrate that bilayer CrBr${}_{3}$ not only has the R-type structures, but also another kind of stacking structure called H-type, in which the two layers are aligned in anti-parallel directions. In fact, the H-type structure can be viewed as one layer of the R-type bilayer having an in-plane 180${}^{\circ }$ rotation. The two types of structures exhibit different interlayer magnetic coupling [5,18]. Inspired by those results, we investigate the H-type bilayer CrI${}_{3}$ by first-principles calculations. It is found that the interlayer magnetic coupling of H-type bilayer CrI${}_{3}$ is stacking-dependent, and is switchable by modulating the out-of-plane strain and electron doping.

## 2. Computational Method

The first-principles calculations are performed in the Vienna Ab initio simulation package (VASP) [19]. The Perdew–Burke–Ernzerhof (PBE) [20] functional for the generalized gradient approximation (GGA) is chosen to describe the exchange–correlation interaction. A plane-wave cutoff energy of 520 eV and a 9×9×1 Monkhorst-Pack k-points sampling are adopted in spin-polarization calculations. The vacuum space along the *z*-axis is set to be 15 Å to eliminate the interaction between adjacent images. The tolerance of force convergence in structure relaxation is 0.01 eV/Å and the energy is converged to $10^{-5}\;\mathrm{eV}$. The on-site Coulomb repulsion of the Cr 3d orbitals is taken into account by using a Hubbard parameter *U* of 3 eV [17,21]. The optB86b functional [22] is used to consider the dispersion interaction, which is verified to be appropriate for vdW materials [15,16,23]. The calculations using other vdW functionals and optB86b functional with spin-orbit coupling (SOC) have also been checked. The carrier doping is implemented by adding electrons or holes into the unit cell and the background-charge is assumed to be homogeneous, which is widely used in the doping simulation and has been proved to be in agreement with experiments [16,24,25].

## 3. Results and Discussion

### 3.1. Geometric Structures

**Figure 1:**
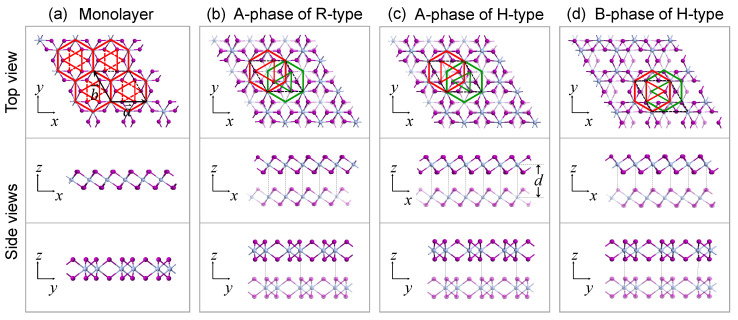
Top and side views of CrI${}_{3}$ structures, in which grey balls represent Cr atoms and purple balls for I atoms. The rhombus of dashed black lines in each subfigure represents a unit cell with the primitive vectors $\vec{a}$ and $\vec{b}$. (**a**) CrI${}_{3}$ monolayer. Six I atoms are surrounded by a hexagon (indicated by solid red lines) composed of six Cr atoms. Three of them are above while the other three are below the plane of Cr atoms, forming two triangles with opposite directions, marked by solid and dashed red lines, respectively. CrI${}_{3}$ bilayers for (**b**) A-phase of R-type, (**c**) A-phase of H-type, and (**d**) B-phase of H-type. Atoms in lower layers are denoted to be translucent, and the hexagons of Cr atoms with the above triangles of I atoms in upper layers and lower layers are indicated by red and green solid lines, respectively. The interlayer distance is defined as *d*.

We start our discussions with the structures of CrI${}_{3}$. Figure 1a shows the top view of the monolayer CrI${}_{3}$, in which Cr atoms are arranged in a honeycomb lattice. Six I atoms are surrounded by a hexagon composed of six Cr atoms, marked by solid red lines. Three of them that are above the plane of Cr atoms form a triangle indicated by solid red lines, and the other three below the plane of Cr atoms form a triangle in the opposite orientation, indicated by dashed red lines. The stacking orientation of the two layers for the R-type CrI${}_{3}$ is illustrated in Figure 1b. As layers are aligned in parallel, it is shown that every triangle formed by the three I atoms on the same side of the Cr atoms plane in a layer points to the same direction. When a layer of the R-type bilayer CrI${}_{3}$ rotates 180${}^{\circ }$ to the other layer, we get the H-type bilayer. It can be observed that the two triangles in the upper and lower layers point to opposite directions in H-type structures, indicating that the two layers are anti-parallel, as shown in Figure 1c,d.

We consider two configurations of H-type, namely the A-phase (Figure 1c) and B-phase (Figure 1d) here. The lateral displacements between the upper and lower layers are distinct in A- and B-phases, showing that the relative positions of the Cr atom hexagons in the upper and lower layers are different. Given that the monolayer CrI${}_{3}$ is intralayer FM coupling with an out-of-plane easy axis, the intralayer spin directions of a layer are set to be uniform in this work. A rhombus of dashed black lines in Figure 1 represents a unit cell with the primitive vectors $\vec{a}$ and $\vec{b}$, and the lattice constant *a* is determined to be 6.90 Å, which is consistent with other experimental and calculating reports [15,16,17].

### 3.2. Interlayer Magnetism and Electronic Structure Properties

#### 3.2.1. Interlayer Magnetic Coupling

We compare the energies of interlayer FM and AFM states in the two phases of H-type bilayer CrI${}_{3}$. The results suggest that the interlayer AFM coupling is more stable ($E_{\mathrm{AFM}}-E_{\mathrm{FM}}<0$) in A-phase, while the B-phase favors the interlayer FM coupling ($E_{\mathrm{AFM}}-E_{\mathrm{FM}}>0$), in H-type structures. As a contrast, the A-phase favors interlayer FM coupling and the B-phase favors interlayer AFM coupling in R-type structures, as shown in Table 1. It implies that rotating a layer of the bilayer CrI${}_{3}$ could modulate the interlayer magnetic coupling. We further check the interlayer distance *d* of the relaxed structures, which is defined as the distance between the planes composed of Cr atoms in the two layers, as illustrated in the side view of Figure 1c. It is found that the A-phase of H-type has a larger interlayer distance than the B-phase and the R-type structures, also shown in Table 1. It indicates that the interlayer magnetic interaction tends to push the two layers away from each other in the A-phase of H-type bilayers.

We also examined whether the magnetic ground state of H-type bilayer CrI${}_{3}$ could be affected by other vdW functionals. In addition, the calculations with optB86b functional including SOC are performed as well. As displayed in Table 2, the A-phase always favors the AFM interlayer coupling while the B-phase always exhibits the FM interlayer coupling. It is verified that the magnetic ground states of bilayer CrI${}_{3}$ are robust to different functionals and insensitive to the including of SOC, which is consistent with other reports [16,17,26].

**Figure 2:**
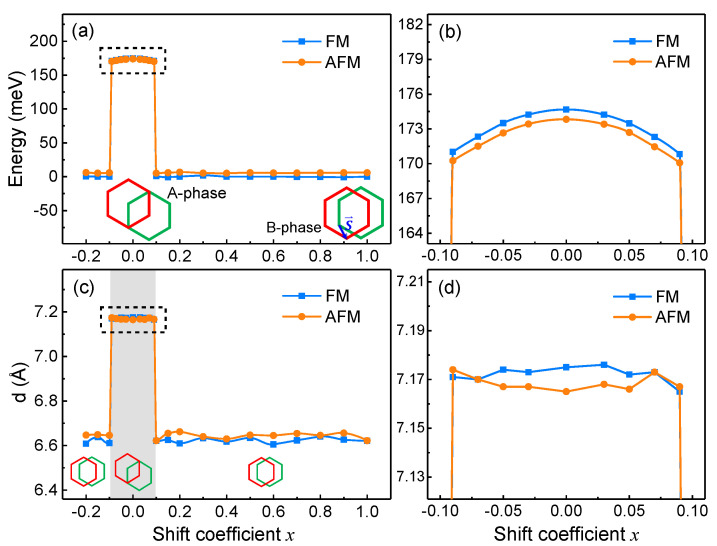
Transition of lateral shift between A-phase and B-phase of H-type bilayer CrI${}_{3}$. Energy of each configuration during the transition is shown in (**a**). The insets illustrate the configurations of A- and B-phases, respectively, and $\vec{S}$ demonstrates the shift pathway of the upper layer from A-phase to B-phase. The displacement in midway is $x\vec{S}$, where *x* is the shift coefficient; (**b**) enlarged view of the dashed box marked in (**a**); (**c**) interlayer distance of each optimized configuration in the transition process. The insets indicate the configuration type in each range of shift coefficient *x*, i.e., A-phase-like in the grey area and B-phase-like in the other areas; (**d**) enlarged view of the dashed box marked in (**c**).

As mentioned above, A-phase and B-phase have different interlayer magnetic coupling, and the interlayer stacking order between the upper and lower layers are different in these two phases. Actually, the upper layer shifts laterally along the vector $\vec{S}$ from the A-phase to the B-phase, where $\vec{S}=-\vec{b}/3$, as shown in the inset of Figure 2a. We study the transition step by step along the direction of $\vec{S}$. The lateral displacement of the upper layer in an intermediate structure is $x\vec{S}$, where *x* is called the shift coefficient. When x=0, the structure is in A-phase; when x=1, the structure is in B-phase. The energies of FM and AFM states with *x* are shown in Figure 2a, and the portion of x∈(−0.1,0.1) is enlarged in Figure 2b. Here, the energy is defined with respect to the energy of the FM state in B-phase. According to the energy curves, only within a small range around A-phase (|x|<0.1), the structures still maintain the interlayer AFM coupling as A-phase, while the other structures turn to the interlayer FM coupling as B-phase. Considering that the energy manifests a step change as the magnetic ground state switches, we infer that it could be due to the structures, which are optimized to different types of phases, and this view is confirmed by examining the relaxed structures. The interlayer distance *d* of each relaxed structure during the transition is shown in Figure 2c, and the portion of x∈(−0.1,0.1) is enlarged again in Figure 2d. When |x|<0.1, the optimized structures remain similar to the A-phase in the stacking order and the large distance between the layers, so they exhibit AFM ground state the same as the A-phase. However, when |x|≥0.1, the interlayer distance suddenly diminishes and the interlayer stacking order turns B-phase-like, leading to their FM interlayer coupling the same as the B-phase.

In other words, the optimized structures could be classified into two types, i.e., the A-phase-like configurations and the B-phase-like ones. Configurations of the same type possess similar stacking orders and the same interlayer magnetic coupling. It indicates that the interlayer magnetic coupling of the H-type bilayer CrI${}_{3}$ is stacking-dependent. We choose A-phase and B-phase as the representatives of the two types of magnetic configurations for subsequent investigations because they are high-symmetry configurations, and these two phases are experimentally manifested to be stable in R-type CrI${}_{3}$ [15].

**Figure 3:**
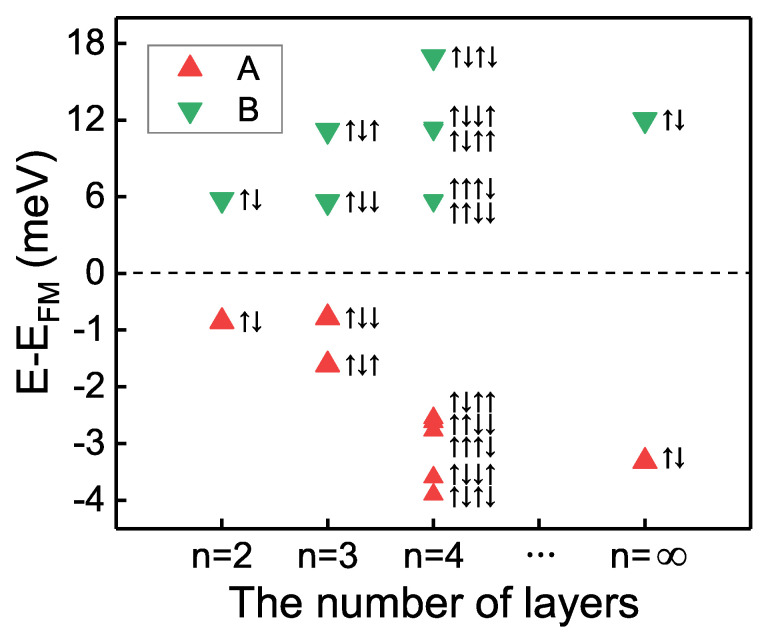
Energies in A- and B-phases for different spin configurations with different layer numbers *n*, including CrI${}_{3}$ bilayer (n=2), trilayer (n=3), quadrilayer (n=4), and bulk (n=∞). The energy of the FM state for the corresponding structure is subtracted from each energy. The magnetization direction of a layer is represented by ↑ or ↓ arrow, indicating spin-up and spin-down, respectively. The magnetic order of each AFM state is represented by a combination of ↑ and ↓ next to the data marker.

We further calculate the interlayer magnetic coupling of multilayer H-type CrI${}_{3}$, in which every two adjacent layers are aligned in anti-parallel directions. For a structure with a certain number *n* of layers, the FM state is the one in which the magnetization directions of all the layers are identical, whereas the structures with layer numbers greater than two (n>2) have more than one AFM state. The interlayer magnetic order of each AFM state is represented by a combination of ↑ and ↓ next to the data marker in Figure 3. To obtain the magnetic ground state, we subtract the energy of the FM state for the corresponding structure from the energy of each AFM configuration. The results imply that, though the number of layers increases from two, three, four, even to infinity (bulk), the energies of the AFM states in A-phase are always lower than that of the FM state, and the configuration with AFM coupling between every two adjacent layers has the lowest energy, while the energy of the FM configuration is always the lowest in B-phase. The conclusion is that the structures of A-phase stacking always maintain the interlayer AFM coupling while the structures of B-phase stacking always favor the interlayer FM magnetic ground state, which is robust to the number of layers.

#### 3.2.2. Electronic Structure Properties

We mainly focus on the H-type bilayer CrI${}_{3}$. The structures mentioned in the following text are H-type CrI${}_{3}$ bilayers unless otherwise specified.

**Figure 4:**
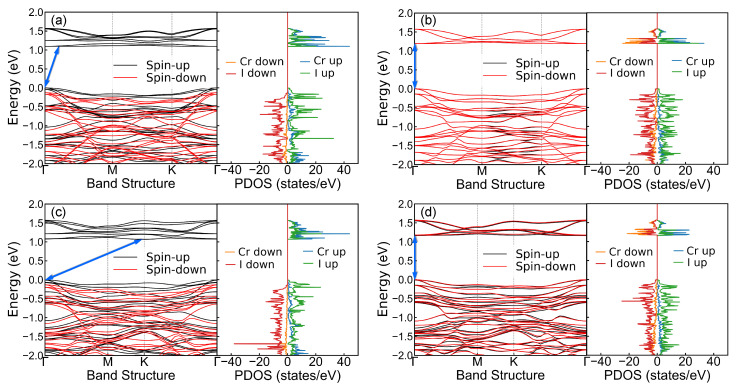
Band structures and PDOS of (**a**) FM state in A-phase; (**b**) AFM state in A-phase; (**c**) FM state in B-phase; (**d**) AFM state in B-phase, for H-type bilayer CrI${}_{3}$. The blue double-headed arrow in each subfigure points out the positions of CBM and VBM.

To investigate the electronic structure properties, we plot the band structures and the partial density of states (PDOS) for A- and B-phases in interlayer FM and AFM states, as shown in Figure 4. It can be seen from Figure 4a,c that the bands of spin-up and spin-down in FM states are split, and the corresponding PDOS of the two spin channels are asymmetrical, which is a manifestation of interlayer FM coupling. However, in the AFM states of Figure 4b,d, the bands of spin-up and spin-down remain degenerate, and the PDOS is almost bilaterally symmetrical, reflecting the interlayer AFM coupling. We figure out that the bandgaps of FM in A-phase, AFM in A-phase, FM in B-phase, and AFM in B-phase are 1.10, 1.20, 1.07, and 1.16 eV, respectively. According to the positions of the conduction band minimum (CBM) and the valence band maximum (VBM) indicated by the blue double-headed arrows in Figure 4, the configurations of FM state in A- and B-phases are indirect bandgap semiconductors, while the configurations of AFM state are direct bandgap semiconductors. It is worth noting that the FM states in both A- and B-phases exhibit half-semiconductivity [27], that is, the valence and conduction bands near the Fermi level are contributed by the same spin channel (spin-up here), leading to 100% spin polarization. This feature is possibly applied to injection and detection of spin-polarized carriers [27,28]. As mentioned above, the magnetic ground states of interlayer coupling are AFM for A-phase and FM for B-phase corresponding to Figure 4b and Figure 4d, respectively, and the two states have different band structures, bandgap types, and electron state distributions. It indicates that changing the stacking order can not only change the interlayer magnetic coupling but also control the electronic structure properties.

### 3.3. Superexchange Mechanism of Magnetism

The magnetism of CrI${}_{3}$ is mainly attributed to the Cr atoms, each of which has three half-occupied $t_{2g}$ orbitals and two empty $e_{g}$ orbitals. Every I atom forms a covalent bond with each of two neighboring Cr atoms, so that the *p* orbital of an I atom couples with the *d* orbitals of two Cr atoms. Since there is an intralayer FM coupling, the spins of Cr atoms inside a layer are aligned in the same direction, and the bonded I atoms are magnetized reversely by Cr atoms. The magnetic coupling of CrI${}_{3}$ originates from the Cr-I-Cr superexchange interaction [29,30], in which the *p* orbital of an I atom serves as an intermedium between the *d* orbitals of two Cr atoms, allowing the electron to hop between different Cr atoms. Due to the strong on-site Coulomb repulsion of the Cr 3d electrons, which is stronger than the orbital splitting energy (i.e., U>Δ), the Cr atoms tend to be in high-spin state. According to the theory proposed by Goodenough and Kanamori [31,32,33], the superexchange will be AFM when the electrons hop between two occupied *d* orbitals ($t_{2g}$-$t_{2g}$), while hopping between one occupied and another empty *d* orbital ($t_{2g}$-$e_{g}$) leads to FM superexchange.

**Figure 5:**
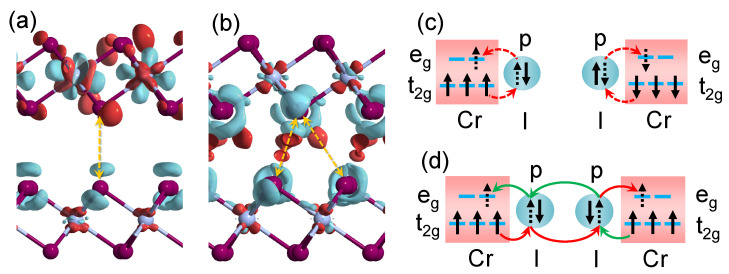
Charge density differences after stacking the upper and lower layers together of (**a**) AFM state in A-phase and (**b**) FM state in B-phase. The red isosurface contours indicate the charge accumulation, and the cyan ones show the charge depletion. The yellow dashed arrows mark out the nearest interacting I atoms between the two layers; (**c**,**d**) schematics of interlayer magnetic coupling in (**a**,**b**), respectively.

To understand the underlying mechanism of the different magnetism of A- and B-phases, we calculate the charge density differences before and after stacking the upper and lower layers together for their configurations of magnetic ground state, i.e., AFM state in A-phase and FM state in B-phase, as shown in Figure 5a and Figure 5b, respectively. The red isosurface contours indicate the charge accumulation, and the cyan isosurface contours show the charge depletion. The isosurface value is set to be 0.001 e/Å${}^{3}$. As can be seen from Figure 5a, no obvious charge accumulation appears in the vdW gap between the two layers in the A-phase, implying that electrons cannot hop between the two layers. The reason should be that the layer spacing of A-phase is large and the distance between the Cr atoms of two layers is far, so the superexchange interaction mainly exists between the intralayer atoms. Since an I atom in the upper layer is directly above another one in the lower layer, as shown with the yellow dashed arrow in Figure 5a, it is conceivably more favorable to release the Pauli repulsion when the I atoms of the two layers have the anti-parallel spins, and the Cr atoms of the two layers will have the anti-parallel spins as well in that case, resulting in interlayer AFM coupling in A-phase, as the schematic in Figure 5c illustrated, in which the red dashed arrows indicate the intralayer electron hopping pathway of each layer.

As for the B-phase, due to the different stacking order, the distances between an I atom in one layer and two adjacent I atoms in the other layer are close to each other, forming two I-I interaction channels, marked by yellow dashed arrows in Figure 5b. Apparently, the charge accumulations appear in the middle of both the channels. It can be considered that there is a virtual site, which allows electrons to stop over in the vdW gap, so that consecutive electron hopping of $t_{2g}$-*p*-*p*-$e_{g}$ between two layers is allowed, through two pathways represented by red and green arrows in Figure 5d respectively. This mechanism leads to the interlayer FM coupling in the B-phase.

### 3.4. Controlling of Interlayer Magnetic Coupling

#### 3.4.1. Effect of Out-of-Plane Axial Strain

**Figure 6:**
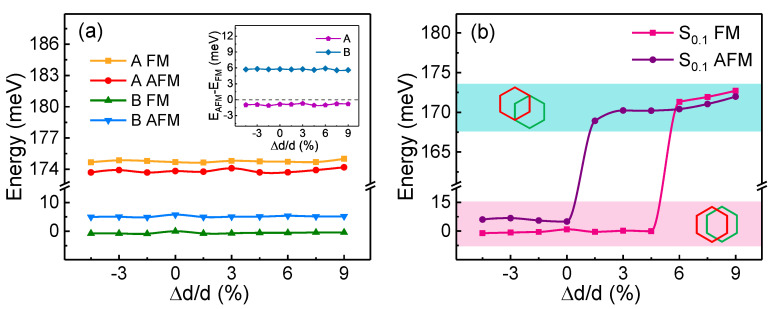
(**a**) Energies of interlayer FM and AFM states in A- and B-phases with different layer distances. The inset is the energy differences between AFM and FM states ($E_{\mathrm{AFM}}-E_{\mathrm{FM}}$) in A-phase and B-phase; (**b**) energies of FM and AFM states for the configuration of which the upper layer laterally shifts $0.1\vec{S}$ from the A-phase (represented by $S_{0.1}$). The optimized structures are B-phase-like in the energy range of the pink area, as illustrated in the lower inset, while the optimized structures are A-phase-like in the energy range of the cyan area, as illustrated in the upper inset. All energies have subtracted the energy of FM state in B-phase without strain.

We examined whether the interlayer magnetic coupling would be affected by the axial strain perpendicular to layer planes as well. According to Figure 6a, the interlayer AFM coupling always has lower energy than FM coupling in A-phase, while the energy of FM coupling is always lower in B-phase, no matter whether the layer distance increases or decreases. That is to say, the magnetic ground states in A-phase and B-phase are too robust to be altered by the out-of-plane axial strain.

In particular, we consider the structure of which the shift coefficient *x* is 0.1 in Figure 2, as it is a turning point of interlayer coupling from AFM to FM. For convenience, we name this structure $S_{0.1}$. It is worth noting that $S_{0.1}$ exhibits interlayer FM coupling in the absence of strain. We plot the energies of $S_{0.1}$ with different layer distances in FM and AFM states respectively, as shown in Figure 6b. It can be seen that the FM state of the structure is more stable when the layer distance is small (Δd/d<6%). When the layer spacing increases by 6%, the energy of the AFM state becomes the lower one, which means that the magnetic ground state changes to the AFM state. By checking the optimized structure, we confirm that it is caused by the difference of relaxed structures. In Figure 6b, the relaxed structures corresponding to the low-energy region (denoted by pink) are B-phase-like, both in terms of the stacking order and the layer distance. However, the relaxed structures corresponding to the high energy area (shown in cyan) have a similar stacking order and layer distance to that of the A-phase. Interestingly, when Δd/d is between 1.5% and 4.5%, the FM and AFM states are optimized to different structures, i.e., B-like phase and A-like phase, respectively, resulting in a large energy difference between them. The mechanism should be that the initial structure of $S_{0.1}$ is actually more similar to the A-phase, as its upper layer only laterally shifts 10% compared to A-phase. In the absence of strain, however, the interlayer interaction forces it to relax into a phase of B-like, including the interlayer stacking order and the interlayer distance, so that the interlayer FM ground state is the same as that of the B-phase. The interaction between layers becomes weaker and weaker as the layer distance increases gradually. When the spacing of layers is large enough (Δd/d≥6%), the optimized structure can remain the A-like phase instead of turning to the B-like phase, which causes the switch of the interlayer magnetic ground state from FM to AFM. The above results imply that the interlayer magnetic coupling of the H-type bilayer CrI${}_{3}$ is switchable by controlling the interlayer stacking order and applying the out-of-plane axial strain.

#### 3.4.2. Effect of Doping

**Figure 7:**
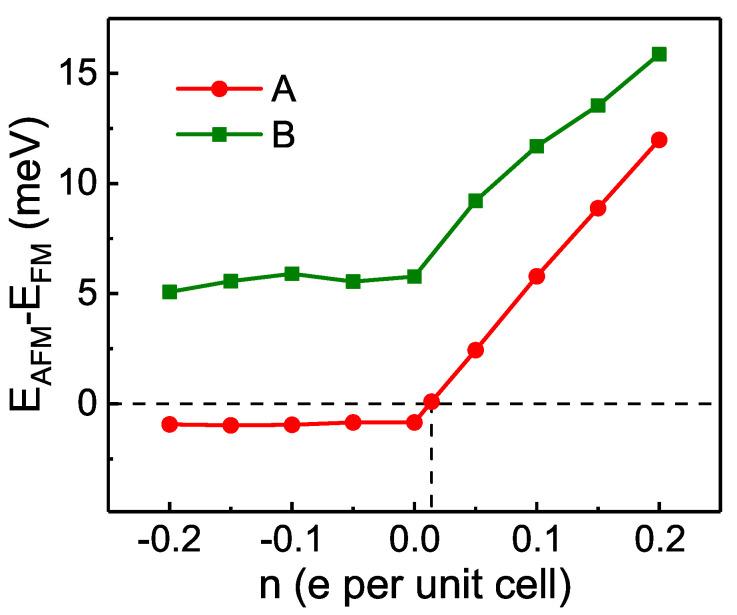
The evolution of energy differences between interlayer AFM and FM states ($E_{\mathrm{AFM}}-E_{\mathrm{FM}}$) of A- and B-phases with different doping concentrations. When the electron doping concentration increases to about 0.014e per unit cell, the magnetic ground state of A-phase turns from AFM to FM.

The effect of doping on the interlayer magnetic coupling is also explored, as shown in Figure 7. In both A- and B-phases, the energy difference between interlayer AFM state and FM state ($E_{\mathrm{AFM}}-E_{\mathrm{FM}}$) varies little with hole doping concentration but tends to rise linearly with the increasing electron doping concentration. For B-phase, $E_{\mathrm{AFM}}-E_{\mathrm{FM}}$ is always negative regardless of doping holes or electrons, indicating the robustness of the interlayer magnetic coupling to doping. As for the A-phase, $E_{\mathrm{AFM}}-E_{\mathrm{FM}}$ turns positive when the electron doping concentration increases to about 0.014e per unit cell, suggesting that the interlayer magnetic ground state switches from AFM to FM. In other words, the interlayer magnetic coupling of A-phase stacking structures for H-type bilayer CrI${}_{3}$ can be controlled by electron doping.

## 4. Conclusions

In summary, we investigate the interlayer magnetic coupling of H-type bilayer CrI${}_{3}$ based on first-principles calculations. We seek out two magnetic coupling states with different electron structure properties, and they are stacking-dependent. The different interlayer magnetism can be attributed to different interlayer superexchange mechanisms. The interlayer magnetic coupling can be turned by applying out-of-plane axial strain or electron doping. Our work provides promising applications in manipulating magnetism of 2D vdW materials, as well as the design of heterostructures and spintronic devices based on 2D magnetic vdW materials.

## Figures and Tables

**Table 1:** Energy differences between interlayer AFM and FM states ($E_{\mathrm{AFM}}-E_{\mathrm{FM}}$), magnetic ground states (MGS), and the interlayer distances (*d*) of H-type bilayer CrI${}_{3}$ in A- and B-phases. For comparison, the data of R-type are also listed.

Configuration		$E_{\mathrm{AFM}}-E{}_{\mathrm{FM}}\;\left(\mathrm{meV}\right)$	MGS	*d*(Å)
H-type	A	−0.851	AFM	7.17
	B	5.780	FM	6.62
R-type	A	10.713	FM	6.61
	B	−1.126	AFM	6.62

**Table 2:** Energy differences between interlayer AFM and FM states ($E_{\mathrm{AFM}}-E_{\mathrm{FM}}$) of H-type bilayer CrI${}_{3}$ in A- and B-phases with different vdWs. The calculations with optB86b and spin-orbit coupling (SOC) are also listed.

$E_{\mathrm{AFM}}-E_{\mathrm{FM}}$ (meV)	DFT-D2	DFT-D3	optPBE	optB88	optB86b	optB86b+SOC
A-phase	−0.360	−0.211	−0.180	−1.512	−0.851	−0.941
B-phase	7.526	5.776	4.056	4.091	5.780	5.555

## Data Availability

The data presented in this study are available on request from the corresponding author.
